# Tudor staphylococcal nuclease is a structure-specific ribonuclease that degrades RNA at unstructured regions during microRNA decay

**DOI:** 10.1261/rna.064501.117

**Published:** 2018-05

**Authors:** Chia-Lung Li, Wei-Zen Yang, Zhonghao Shi, Hanna S. Yuan

**Affiliations:** 1 Institute of Molecular Biology, Academia Sinica, Taipei, Taiwan 11529, ROC; 2 Graduate Institute of Biochemistry and Molecular Biology, National Taiwan University, Taipei, Taiwan 10048, ROC

**Keywords:** ribonuclease, microRNA decay, RNA editing, RNA silencing, TSN

## Abstract

Tudor staphylococcal nuclease (TSN) is an evolutionarily conserved ribonuclease in eukaryotes that is composed of five staphylococcal nuclease-like domains (SN1–SN5) and a Tudor domain. TSN degrades hyper-edited double-stranded RNA, including primary miRNA precursors containing multiple I•U and U•I pairs, and mature miRNA during miRNA decay. However, how TSN binds and degrades its RNA substrates remains unclear. Here, we show that the *C. elegans* TSN (cTSN) is a monomeric Ca^2+^-dependent ribonuclease, cleaving RNA chains at the 5′-side of the phosphodiester linkage to produce degraded fragments with 5′-hydroxyl and 3′-phosphate ends. cTSN degrades single-stranded RNA and double-stranded RNA containing mismatched base pairs, but is not restricted to those containing multiple I•U and U•I pairs. cTSN has at least two catalytic active sites located in the SN1 and SN3 domains, since mutations of the putative Ca^2+^-binding residues in these two domains strongly impaired its ribonuclease activity. We further show by small-angle X-ray scattering that rice osTSN has a flexible two-lobed structure with open to closed conformations, indicating that TSN may change its conformation upon RNA binding. We conclude that TSN is a structure-specific ribonuclease targeting not only single-stranded RNA, but also unstructured regions of double-stranded RNA. This study provides the molecular basis for how TSN cooperates with RNA editing to eliminate duplex RNA in cell defense, and how TSN selects and degrades RNA during microRNA decay.

## INTRODUCTION

Tudor staphylococcal nuclease (TSN; also known as Tudor-SN, SND1, or p100) is a conserved multifunctional protein in eukaryotes that participates in various cellular events, from transcriptional to post-transcriptional regulation. TSN was first identified as a transcription coactivator interacting with transcription factors such as EBNA-2, cMyb, STAT6, and STAT5 to promote cell proliferation and differentiation in mammals (Leverson et al. 1998; Abe et al. 2005; Välineva et al. 2005; Paukku et al. 2007). Subsequently, a positive correlation between carcinogenesis and TSN overexpression was discovered in different types of cancers, including colon cancer (Tsuchiya et al. 2007), hepatocellular carcinoma (Yoo et al. 2011), breast cancer (Blanco et al. 2011; Wan et al. 2014), prostate cancer (Kuruma et al. 2009), and malignant glioma (Emdad et al. 2015). Moreover, TSN is cleaved by caspases and facilitates programmed cell death during development and stress responses (Sundström et al. 2009). TSN also interacts with small ribonucleoproteins and Sm proteins involved in pre-mRNA splicing (Yang et al. 2007; Gao et al. 2012; Cappellari et al. 2014). In plants, TSN is an RNA-binding protein involved in RNA transport and localization, which is essential for stress tolerance and has been linked to the formation of stress granules and processing bodies (Wang et al. 2008; Frei dit Frey et al. 2010; Gutierrez-Beltran et al. 2015). Hence, TSN functions as a scaffold for protein–protein or protein–RNA interactions in transcription regulation, mRNA localization, and pre-mRNA splicing (Gutierrez-Beltran et al. 2016).

Besides functioning as a scaffold protein, TSN also possesses ribonuclease activity linked to microRNA (miRNA) decay. TSN was first identified as a component of the RNA-induced silencing complex (RISC) in *Caenorhabditis elegans*, *Drosophila,* and mammals (Caudy et al. 2003); although Argonaute 2, and not TSN, is the “slicer” in RISC responsible for siRNA-guided mRNA degradation during RNA silencing. This finding prompted the question as to which types of RNA are cleaved by TSN in vivo. A series of interesting studies have shown that *X. laevis* and human TSN degrades hyper-edited double-stranded RNA (dsRNA) containing multiple I•U and U•I pairs generated by ADAR (adenosine deaminase acting on RNA), which converts adenosines to inosines in dsRNA (Scadden and Smith 2001; Scadden 2005). TSN thus likely cooperates with RNA editing to dispose of hyper-edited viral RNA in cell defense. Moreover, some primary miRNA precursors, including the precursor of pri-miR142, are edited by ADAR and these edited RNA are further degraded by TSN in mice, suggesting that TSN plays a role in miRNA decay (Yang et al. 2006). Recently, TSN was further identified to be a ribonuclease that degrades both protein-free and Argonaute 2-loaded mature miRNA that promotes the G1/S phase transition in human cells (Elbarbary et al. 2017b). TSN, therefore, plays an important role in miRNA decay in mammals, targeting not only hyper-edited miRNA precursors, but also mature miRNA.

**FIGURE 1.**
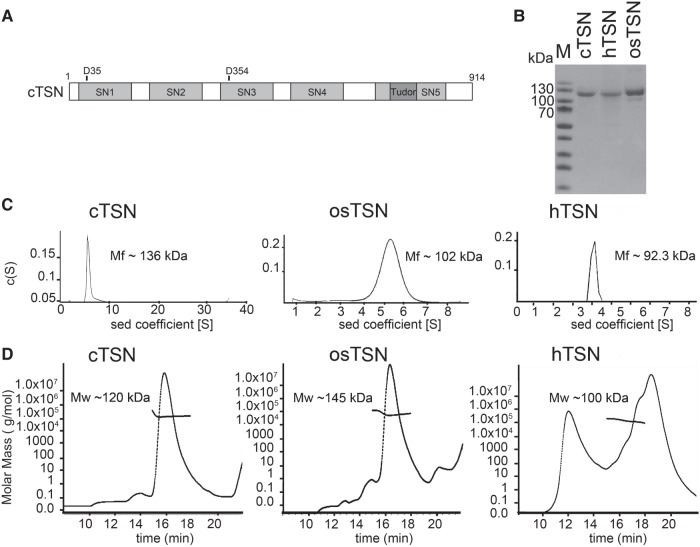
Full-length TSN is a monomeric protein. (*A*) The domain structure of *Caenorhabditis elegans* cTSN. The two putative Ca^2+^-binding residues in the SN1 and SN3 domains are shown at the *top*. (*B*) The SDS–PAGE of the purified recombinant cTSN from *Caenorhabditis elegans*, hTSN from *Homo sapiens*, and osTSN from *Oryza sativa.* (*C*) The molecular weights of cTSN, osTSN, and hTSN were estimated by ultracentrifugation. (*D*) TSN proteins were subjected to SEC–MALS (size-exclusion chromatography coupled with multiangle light scattering) and are represented as elution profiles.

TSN is composed of four tandem repeats of staphylococcal nuclease-like domains (SN1-4), followed by an SN5 domain incorporating an inserted Tudor domain (Fig. 1A; Callebaut and Mornon 1997). The four tandem SN1-4 domains are responsible for RNA binding and cleavage (Li et al. 2008), whereas the Tudor domain interacts with symmetrically dimethylated arginines of small nuclear ribonuceloprotein (snRNP) or Piwi family proteins through its conserved aromatic cage (Shaw et al. 2007; Friberg et al. 2009; Liu et al. 2010; Musiyenko et al. 2012). A crystal structure of the C terminal of human TSN (hTSN) that included four domains (SN3–SN4–Tudor–SN5) revealed a basic concave surface in the SN3–SN4 region that is likely involved in RNA binding (Li et al. 2008). A crystal structure of the N-terminal SN1–SN2 domain suggests a unique groove between the SN1 and SN2 domains that binds to the partner protein, Metadherin, and has been linked to the development of breast cancer (Guo et al. 2014). Thus, TSN harbors multiple domains that allow it to play various roles in RNA and protein interactions.

Compared to its bridging roles in protein–protein interactions, how TSN selects its RNA substrates for cleavage is less clear. TSN specifically degrades duplex RNA with I•U and U•I pairs, which are particularly unstable since duplex RNA containing single or double I•U/U•I pairs have much lower thermal stability compared to those with Watson–Crick base pairs (Serra et al. 2004). This finding prompted us to examine if TSN cleaves dsRNA with mismatched base pairs. Here, we show that *Caenorhabditis elegans* TSN (cTSN) degrades both ssRNA and mismatched dsRNA, but not perfectly matched dsRNA. Therefore, TSN targets the unstructured regions of RNA, but not necessarily those with inosines or I•U/U•I pairs. We also identified multiple ribonuclease active sites in the SN1 and SN3 domains of cTSN and show by small-angle X-ray scattering (SAXS) that rice TSN (osTSN) has a flexible two-lobed conformation. Together, our results reveal how TSN selects its targets for degradation and explain how TSN degrades inosine-edited miRNA precursors and mature miRNA during miRNA decay.

## RESULTS

### TSN is a monomeric protein

We first inserted the cDNA encoding TSN from worms (*Caenorhabditis elegans*), plants (*Oryza sativa*), and mammals (*Homo sapiens*) into the expression vectors to express TSN proteins with an N-terminal His-tag in *E. coli*. Human, rice, and *C. elegans* TSN, named hTSN, osTSN, and cTSN, respectively, were purified by chromatography with a high homogeneity (Fig. 1B). The molecular weights of TSN were estimated by analytical ultracentrifugation (AUC): 136 kDa for cTSN (calculated MW: 101 kDa), 102 kDa for osTSN (calculated MW: 108 kDa), and 92 kDa for hTSN (calculated MW: 102 kDa) (Fig. 1C). Similar molecular weights—120 kDa for cTSN, 145 kDa for osTSN, and 100 kDa for hTSN—were estimated for TSN proteins using SEC–MALS (size-exclusion chromatography coupled with multiangle light scattering) (Fig. 1D). These results suggest that these three recombinant TSN proteins are monomeric in solution. However, hTSN and cTSN were expressed at low levels in *E. coli*, and hTSN was highly unstable, tending to decay and aggregate (see Fig. 1D). In contrast, osTSN was highly expressed and could be purified in large quantities. As a result, we continued our RNA degradation assays using cTSN since it shares a higher sequence identity of 47% with hTSN, but performed SAXS (small-angle X-ray diffraction) using osTSN, which has a lower sequence identity of 33% with hTSN.

### cTSN is a Ca^2+^-dependent RNase cleaving at the 5′-side of a phosphodiester bond

**FIGURE 2.**
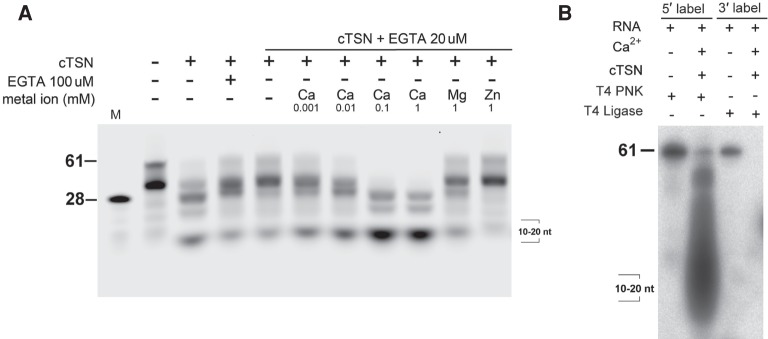
cTSN is a Ca^2+^-dependent endonuclease cleaving at the 5′-side of phosphodiester bonds. (*A*) cTSN (100 nM) degraded the 5′-fluorescein-labeled pre-miR142 RNA (500 nM) in the presence of Ca^2+^ at concentrations of 0.1–1 mM. The sizes of pre-miR142 (68 nt) and an RNA marker (28 nt) were labeled in the *left* of the gel. (*B*) cTSN cleaved at the 5′-side of phosphodiester bonds to produce degraded fragments with 3′-phosphate and 5′-OH ends that could be labeled by T4 polynucleotide kinase (T4 PNK) but not by T4 RNA ligase (T4 ligase).

As the endonuclease activity of hTSN is promoted by calcium ions (Elbarbary et al. 2017b), we first examined the metal ion-dependent activity of cTSN. We prepared a stem–loop RNA, named pre-miR142, which shares a similar structure and sequence to the pre-miR142 precursor that was used in a previous report (Yang et al. 2006). cTSN was incubated with the 5′-end fluorescein-labeled pre-miR142 in buffers containing different metal ions, and the digested RNA fragments were analyzed by gel electrophoresis (Fig. 2A). The purified cTSN could degrade RNA before metal ions were added and this activity was inhibited by 100 µM EGTA, suggesting that the recombinant cTSN was bound with endogenous metal ions (see the first three lanes in Fig. 2A). To remove the endogenous metal ions, cTSN was pre-treated with 20 µM EGTA prior to the RNA degradation assays. The EGTA-treated cTSN had almost no RNase activity, suggesting that the endogenous metal ions had been mostly removed. Addition of calcium ions promoted cTSN endonuclease activity at optimal concentrations of 0.1 to 1 mM (Fig. 2A). However, cTSN had only residual endonuclease activity with 1 mM magnesium ions and no activity with 1 mM zinc ions. Together, these results suggest that cTSN is a Ca^2+^-dependent endonuclease in RNA degradation, similar to the bacterial homolog staphylococcal nuclease (Cotton et al. 1979).

To examine the products cleaved by cTSN, the digested RNA was further incubated with two different end-labeling enzymes: T4 polynucleotide kinase (T4 PNK) that only adds P^32^-labeled-phosphates to 5′ hydroxyl groups of RNA chains, and T4 RNA ligase that only adds P^32^-labeled-phosphates to the 3′ hydroxyl groups. The labeling results clearly show that the cleaved RNA fragments could only be labeled by T4 PNK and not by T4 ligase, suggesting that the digested products had 5′-hydroxyl and 3′-phosphate ends (Fig. 2B). In summary, cTSN is a Ca^2+^-dependent ribonuclease that conducts endonucleolytic cleavage at the 5′ site of the phosphodiester bonds of RNA chains.

### cTSN preferentially binds double-stranded RNA harboring inosines

**FIGURE 3.**
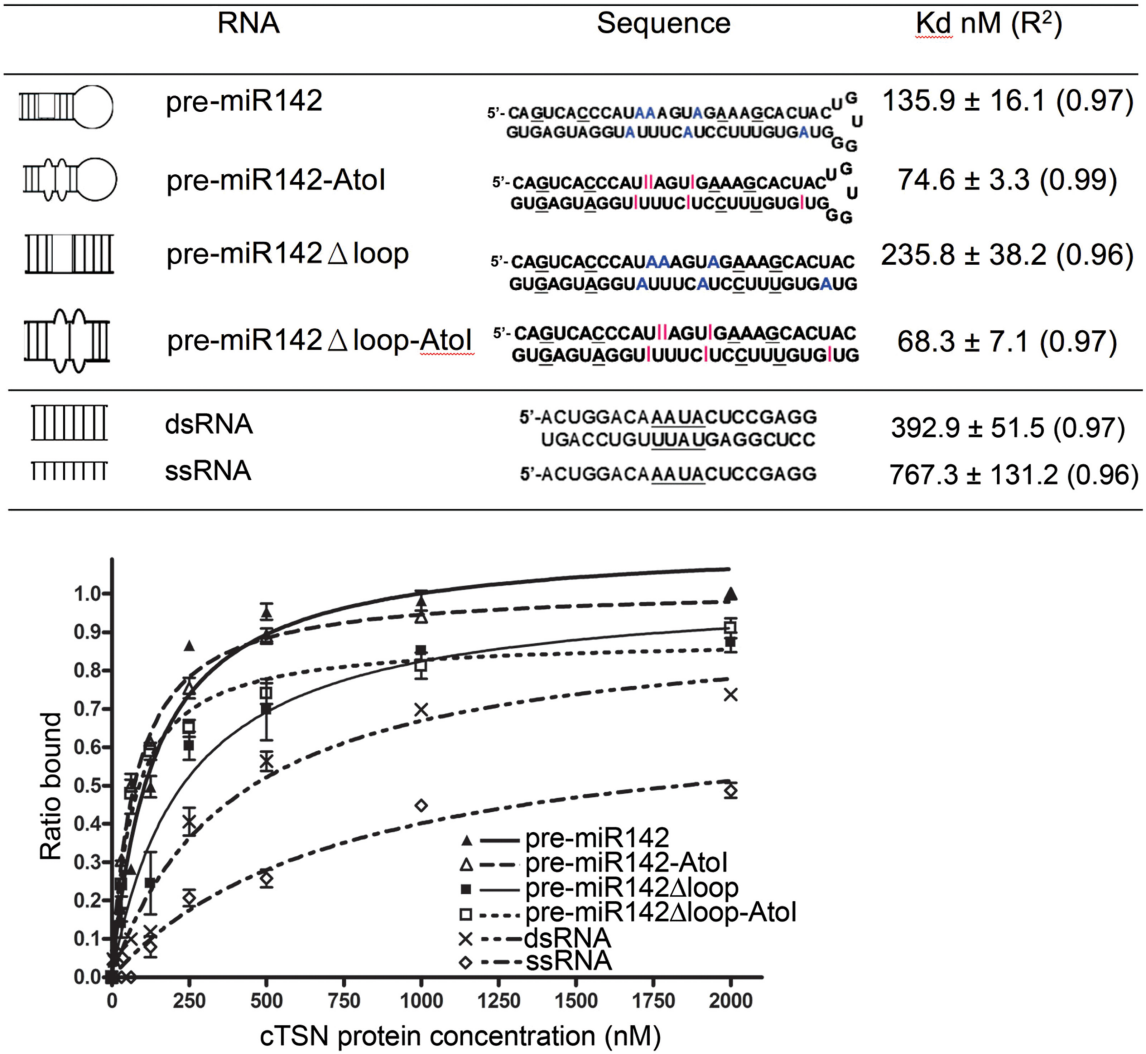
cTSN preferentially binds double-stranded RNA containing I•U and U•I pairs. cTSN (0–2.0 µM) was incubated with 5′-P^32^-labeled RNA for filter-binding assays. Binding percentages from three measurements were calculated to derive the apparent *K*_d_ values between cTSN and RNA by a one-site binding curve, with *R*^2^ (all greater than 0.95) shown in parentheses.

Human TSN degrades A-to-I-edited primary miRNA precursors, including pre-miR142 (Yang et al. 2006). Next, we tested if cTSN preferentially binds to the A-to-I-edited pre-miR142 by filter-binding assays. We prepared a 5′-end P^32^-labeled stem–loop RNA mimicking the primary miR142 precursor but in which the Dicer-cleavable loop region was replaced with a short loop (referred to as pre-miR142), as well as its inosine-edited counterpart (termed pre-miR142-AtoI) (see the RNA sequences in Fig. 3). We found that A-to-I editing indeed improved the binding affinity between cTSN and RNA, with a twofold greater *K*_d_ of 74.6 ± 3.3 nM for the edited pre-miR142-AtoI compared to a *K*_d_ of 135.9 ± 16.1 nM for the unedited pre-miR142. We also generated unedited and edited RNA lacking the short loop region, named pre-miR142Δloop and pre-miR142Δloop-AtoI, respectively. A-to-I editing again increased the *K*_d_ value (about fourfold) of cTSN and RNA-binding affinity; a *K*_d_ of 235.8 ± 38.2 nM for unedited pre-miR142Δloop and 68.3 ± 7.1 nM for edited pre-miR142Δloop-AtoI, respectively. cTSN bound the RNA with the loop (pre-miR142) more strongly (twofold) than the RNA lacking the loop (pre-miR142Δloop) (Fig. 2). Taken together, these results show that A-to-I editing increased binding affinity two- to fourfold, whereas the 5-nucleotide (nt) loop region increased the binding affinity of RNA to cTSN twofold.

To further clarify if cTSN prefers to target the ssRNA or ssRNA region imbedded in dsRNA, we also measured the binding affinity between cTSN and a 20-bp perfectly matched dsRNA (dsRNA) versus a 20-nt ssRNA (ssRNA). cTSN bound the dsRNA with an approximately twofold higher affinity (392.9 ± 51.5 nM) than the ssRNA (767.3 ± 132 nM), suggesting that cTSN prefers to bind dsRNA than ssRNA (see Fig. 3). Taken together, these results suggest that cTSN preferentially binds double-stranded RNA possessing inosines or loops.

### cTSN preferentially cleaves ssRNA and the unstructured regions of dsRNA

**FIGURE 4.**
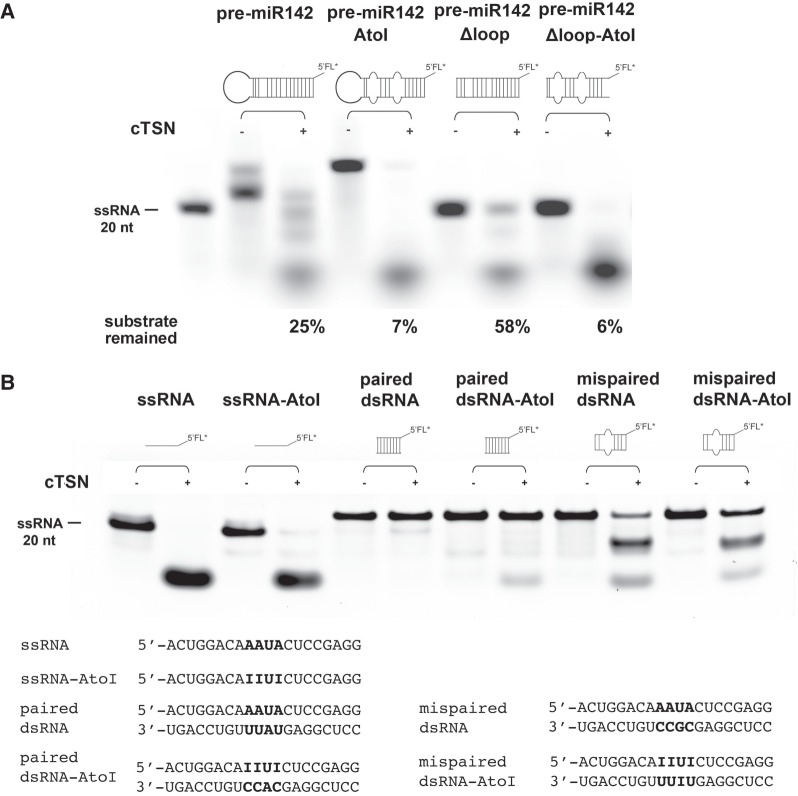
cTSN degrades single-stranded RNA and mismatched double-stranded RNA with or without inosines. (*A*) cTSN (100 nM) degraded stem–loop 5′-fluorescein-labeled RNAs (500 nM) containing mispaired or loop regions (the percentages of the remained substrates are listed at the *bottom* of the gel). (*B*) cTSN (100 nM) degraded single-stranded RNA (500 nM) with or without inosines, as well as double-stranded RNA containing mispaired base pairs, including AAUC/CGCC and IIUI/UIUU. In contrast, cTSN could not degrade paired double-stranded RNA with or without inosines. The RNA sequences are listed at the *bottom* of the gel.

Next, we wondered if cTSN preferentially degrades A-to-I-edited RNA. We incubated the 5′-end fluorescein-labeled pre-miR142 with cTSN and the RNA cleavage patterns were revealed by gel electrophoresis (see Fig. 4A). cTSN degraded the edited pre-miR142AtoI (7% substrate remained) better than its unedited counterpart (pre-miR142, 25% substrate remained). Similarly, cTSN degraded the edited pre-miR142Δloop-AtoI (6% substrate remained) better than the unedited pre-miRNA142Δloop (58% substrate remained). These results are consistent with those of our binding assays and show that inosine editing of miRNA precursors indeed enhances degradation by cTSN.

A-to-I editing introduces unstable I•U or U•I base pairs, suggesting that this type of base-pairing induces structural changes (Serra et al. 2004). Therefore, we set out to examine if cTSN recognizes inosines or mismatched regions. We prepared 20-nt unedited and edited RNAs, referred to, respectively, as ssRNA and ssRNA-AtoI, containing either AAUA or IIUI in the middle of the chain, i.e., similar to those used in a previous analysis (Scadden 2005). We also prepared perfectly paired 20-bp dsRNAs, referred to as “paired dsRNA” and “paired dsRNA-AtoI,” containing either A•U base pairs (AAUA/UAUU) or I•C base pairs (IIUI/CACC) in the middle of the duplex respectively, since inosine (I) and cytosine (C) can form a stable I•C base pair (Watkins and SantaLucia 2005). Our degradation assays show that cTSN degraded ssRNA with or without inosines (i.e., ssRNA and ssRNA-AtoI), but could not degrade the perfectly paired dsRNAs with or without inosines (i.e., paired RNA or paired dsRNA-AtoI) (see Fig. 4B). These results demonstrate that the presence of inosines in RNA is not the determining factor for RNA degradation by cTSN.

We next prepared two mispaired dsRNAs with or without inosines in the middle of the chains: “mispaired dsRNA” containing AAUA/CGCC, and “mispaired dsRNA-AtoI” containing IIUI/UIUU. Notably, cTSN degraded both mispaired dsRNAs, i.e., with or without inosines (Fig. 4B). These findings suggest that, besides ssRNA, cTSN preferentially cleaves dsRNA containing mismatched regions, whether or not they contain inosines. Since A-to-I editing alters the stability of base-pairing in dsRNA, we conclude that the enhanced degradation of inosine-edited RNA by TSN is due to structural changes in the RNA. In summary, our results suggest that cTSN is not an inosine-specific ribonuclease, but instead it preferentially degrades ssRNA and dsRNA containing unstructured mismatched regions.

### Critical catalytic residues in cTSN are located in the SN1 and SN3 domains

**FIGURE 5.**
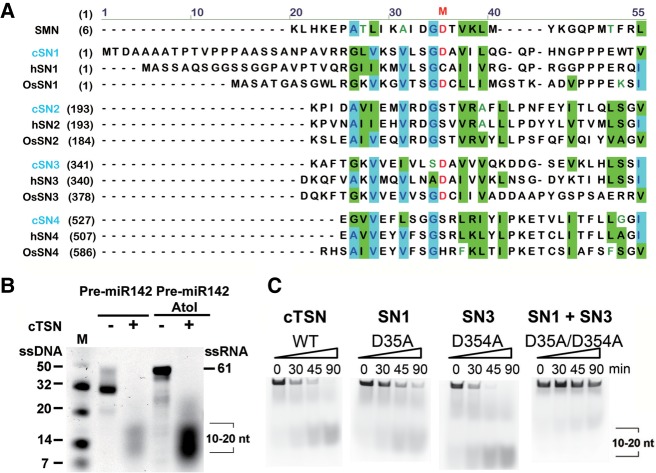
The catalytic residues of cTSN are located in the SN1 and SN3 domains. (*A*) Sequence alignment of the SN1, SN2, SN3, and SN4 domains of cTSN from *Caenorhabditis elegans*, osTSN from *Oryza sativa*, and hTSN from *Homo sapiens*. The putative metal ion-binding residues (M) are marked in red. (*B*) With long degradation time (3 h), cTSN cleaved the RNA substrates, pre-miR142 and pre-miR142AtoI, into small RNA fragments of about 10–20 nt. (*C*) cTSN and its mutants D35A, D354A, and D35A/D354A (100 nM) were incubated with 5′-fluorescein-labeled pri-mR142-AtoI (500 nM). Wild-type cTSN, D35A, and D354A degraded the RNA substrate. The cTSN double mutant D35A/D354A exhibited greatly reduced ribonuclease activity.

Structure-based sequence alignment shows that most putative catalytic residues are mutated in the SN domains of TSN when compared with those of staphylococcal nuclease (Li et al. 2008). The metal ion-binding residues in domains SN2, SN4, and SN5 of TSN proteins are degenerated, whereas one of the calcium-binding residues is conserved in SN1 (Asp35) and SN3 (Asp354) in cTSN (Fig. 5A). To clarify which SN domain is responsible for cTSN's endonuclease activity, we constructed single-point mutants of cTSN, D35A, and D354A, and the double-point mutant D35A/D354A for endonuclease activity assays. With long degradation time (3 h), the wild-type cTSN cleaved pre-miR142 and pre-miR142AtoI into small RNA fragments of about 10–20 nt (Fig. 5B). The single-point mutants D35A and D354A degraded the 5′-end fluorescein-labeled inosine-containing pre-miR142-AtoI comparably to wild-type cTSN (Fig. 5C). In contrast, the double-point mutant D35A/D354A exhibited dramatically reduced endonuclease activity in our time-course assays (Fig. 5C). These results suggest that inactivation of one of the SN domains cannot impair cTSN activity, with the protein likely having at least two active sites in the SN1 and SN3 domains. Our findings agree with the sequence alignment results, which suggest that SN1 and SN3 likely bear endonuclease activities (Li et al. 2008). However, they are not consistent with a truncation study showing that SN1 and SN4 in hTSN are crucial for endonuclease activity (Elbarbary et al. 2017b) (see Discussion).

### Overall structure of TSN

**FIGURE 6.**
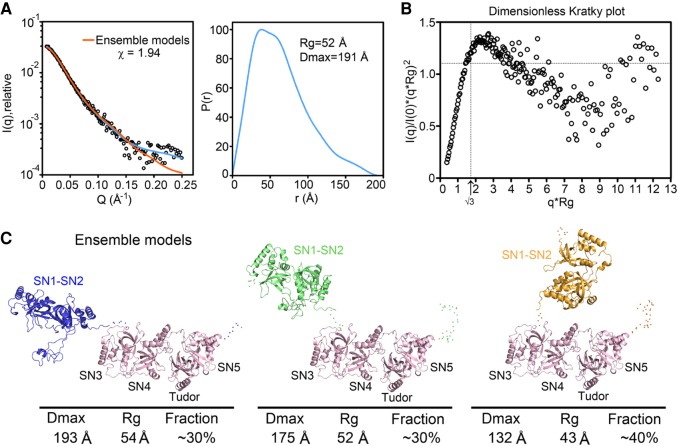
Solution SAXS analysis of rice osTSN reveals a flexible two-lobed structure. (*A*) Small-angle X-ray scattering (SAXS) curves are represented as logarithmic scattering intensities. Theoretical scattering intensities generated from ensemble models are fitted to experimental data with a χ value of 1.94. *Right* panel is the distance distribution function with a maximum diameter (*D*_max_) of 191 Å and a radius of gyration (*R*_g_) of 52 Å. (*B*) The *R*_g_-based dimensionless Kratky plot with curve peak at a *q* × *R*_g_ of greater than √3, indicating flexible conformations. (*C*) Three major ensemble models, with the number of times each conformation existed within the ensemble represented as fractions (%) *below* each conformation.

Although the crystal structures of SN1–SN2 and SN3–SN4–Tudor–SN5 of hTSN were previously reported (Li et al. 2008; Guo et al. 2014), it remains unclear how full-length TSN folds and binds to RNA. Therefore, we performed SAXS (small-angle light scattering) to determine the overall folding of TSN in solution. Only osTSN produced SAXS data of a reasonable quality, but the distance distribution functions show characteristic smoothing toward 0 as *r* approaches *D*_max_ around 191 Å (Fig. 6A), suggesting a flexible elongated conformation as reported previously from FRET analysis (Guo et al. 2014). We calculated the *R*_g_-based dimensionless Kratky plot to reveal that osTSN exhibits some degree of flexibility (Fig. 6B; Receveur-Brechot and Durand 2012). We next modeled the structure of osTSN by the ensemble optimization method (Petoukhov et al. 2012) using the two crystal structures of SN1–SN2 (Guo et al. 2014) and SN3–SN4–Tudor–SN5 (Li et al. 2008) as two rigid bodies in the ensemble search. Three major osTSN conformations were generated and the ensemble gave a fitting curve to the experimental SAXS data with an χ value of 1.94 (Fig. 6A). Two conformations, each representing ∼30% of the ensemble structures, exhibited a “wide open” format with a *D*_max_ of 193 Å and a “half open” conformation with a *D*_max_ of 175 Å, respectively. One conformation, representing ∼40% of the structures, exhibited a “closed” conformation with a *D*_max_ of 132 Å (Fig. 6C). Thus, our SAXS analyses demonstrate that osTSN exhibits a flexible two-lobed conformation in which SN1–SN2 can switch from an open to a closed conformation relative to SN3–SN4–Tudor–SN5. In summary, our SAXS results suggest that TSN has a flexible elongated structure that may change conformation upon RNA binding.

## DISCUSSION

In this study, we show that cTSN is a Ca^2+^-dependent ribonuclease, degrading not only single-stranded RNA but also dsRNA containing mismatched regions arising from A-to-I editing that produces multiple I•U and U•I pairs or from mismatched base-pairing, such as the A•C and U•G pairs used here. Perfectly paired dsRNA substrates with or without inosines are resistant to cTSN cleavage. An inosine-specific ribonuclease (Endo V) has previously been characterized (Morita et al. 2013), and we conclude that TSN is not inosine-specific but is a structure-specific ribonuclease that targets single-stranded RNA and dsRNA with loose regions. Presence of long perfect dsRNA molecules in the cell usually indicates viral infection and invading nucleic acids. TSN may work with ADAR that hyper-edits long dsRNA so that these molecules can be further degraded by TSN in cellular defense (Samuel 2011).

Another important function of TSN is miRNA decay. It has been shown that TSN degrades certain hyper-edited primary miRNA precursors (Yang et al. 2006), as well as a group of mature miRNAs with a preference to cleave at UA and CA dinucleotides (Elbarbary et al. 2017b). Inosine editing may loosen the base-pairing of miRNA precursors and increase their exposure to degradation by TSN. A recent study further shows that TSN interacts with UPF1 helicase which can dissociate miRNA from their mRNA targets, making the mature single-stranded miRNAs susceptible to the TSN-mediated miRNA decay (TumiD) (Elbarbary et al. 2017a). Our results thus reveal how TSN degrades inosine-edited primary miRNA precursors, as well as mature miRNAs, playing an important role in miRNA decay.

TSN is a highly conserved protein and contains five staphylococcal nuclease-like (SN) domains. However, compared with its bacterial homolog, most catalytic residues in the SN domains are degenerated. In this study, we show that *C. elegans* cTSN contains at least two active sites located in domains SN1 and SN3. Interestingly, the putative Ca^2+^-binding residue Asp354 in the SN3 domain is strictly conserved among TSN proteins, but Asp35 in the SN1 domain is only conserved in protozoa, plants, and invertebrates and not in vertebrates. A recent study has shown that the SN1 and SN4 domains in hTSN are important for TSN's catalytic activity (Elbarbary et al. 2017b). As SN1–SN2 and SN3–SN4 are assembled, respectively, into a structural lobe (see Fig. 6), deletion of SN1 or SN4 might disrupt the folding of each lobe and impair TSN's ribonuclease activity, similar to our results of mutations in SN1 and SN3 domains. Further investigations are required to identify the active SN domains in human TSN, which will be important for designing TSN inhibitors in anti-cancer therapy.

## MATERIALS AND METHODS

### Protein expression and purification

The full-length cDNA of *Caenorhabditis elegans* was cloned by RT-PCR. The cDNA of *Homo sapiens* TSN was purchased from OpenBiosystems, whereas the cDNA of *Oryza sativa* TSN was purchased from the KOME database. The cDNA of each TSN was inserted into pET28a expression vector using XhoI and NdeI restriction cutting sites to express proteins with C-terminal 6xHistidine tags in *E. coli*. Site-directed mutagenesis constructs of cTSN were prepared by Pfu-Ultra II Fusion HS DNA Polymerase kit (Agilent Technologies). All constructs were expressed in *E. coli* BL21 (DE3) RIPL cells (Agilent Technologies) at an OD_600_ of 0.6 with 0.5 mM IPTG for 16 h at 18°C. Cells were harvested and lysed at 4°C in 50 mM HEPES (pH 7.0), 150 mM NaCl, 10 mM imidazole, 10% glycerol, 0.1 mM PMSF, 5 mM β-Mercaptoethanol, and 0.1% CHAPS by microfluidizer (Microfluidics M-110P). Cell lysate was passed through a His-Trap HP column (GE HealthCare) and TSN proteins were eluted with 50 mM HEPES (pH 7.0), 150 mM NaCl, 250 mM imidazole, and 5% glycerol. TSN protein samples were then passed through a HiTrap Heparin HP column (GE HealthCare) and eluted with a gradient of 0 to 1 M NaCl in PBS buffer (pH 7.4). Eluted protein samples were adjusted to a concentration of 2 mg/mL in PBS buffer (pH 7.4) with addition of 150 mM NaCl as stock for further experiments.

### Ultracentrifugation

For AUC assays, the recombinant TSN proteins were concentrated to an OD_280_ of 1.0 in 50 mM HEPES (pH 7.0), 150 mM NaCl, 10 mM imidazole, and 1 mM β-mercaptoethanol. Sedimentation velocities of TSN were measured at 40,000 rpm (Beckman XL-A) at 4°C. Multiple scans (OD_280_) at different time intervals were then fitted to a continuous c(s) distribution model using the SEDFIT program.

### SEC–MALS

TSN (1 mg/mL, 100 µL injection volume) was applied to an Agilent-Bio SEC-3 column (Agilent) connected to a DAWN HELIOS II-18 angle MALS (Wyatt Technology) detector with a wavelength setting at 658 nm. Samples were run in a PBS (pH 7.4) buffer containing 150 mM NaCl and 5 mM β-mercaptoethanol, with a flow rate of 0.2 mL/min using the ÄKTA-UPC 900 FPLC system (GE Healthcare). A 2 mg/mL sample of BSA (Sigma Lot#SLBH1159V) was used for calibration. Scattering data were analyzed using the ASTRA 6 software (Wyatt Technology).

### RNA terminal labeling experiments

The pre-miR142 degraded by cTSN was used for terminal labeling assays. For 5′-end labeling, degraded RNA fragments, γ-P^32^-ATP and T4 polynucleotide kinase (Roche) were mixed and incubated at 37°C for 30 min. For 3′-end labeling, degraded RNA fragments, α-P^32^ATP and T4 RNA ligase (NEB) were mixed and incubated at 37°C for 30 min. Reactions were stopped by sample loading buffers and the labeling results were analyzed on 15% TBE–PAGE.

### Filter-binding assays

The 5′-end ^32^P-labeled RNA substrates shown in Figure 3 (purchased from Dharmacon) were incubated with a serial dilution of cTSN (0 to 2 μM) in binding buffer containing 50 mM HEPES (pH 7.0), 250 mM NaCl, 5 mM β-Mercaptoethanol and 2 mM EDTA for 30 min at room temperature. The cTSN–RNA complex solutions were passed through a filter-binding assay device (Bio-Dot SF microfiltration apparatus, Bio-Rad). The isotope signals from cTSN–RNA complex bound on the nitrocellulose membrane and the free RNA passed through the filter-binding assay apparatus were exposed to a phosphorimaging plate and visualized by autoradiography (Fujifilm, FLA-5000). The intensities of cTSN–RNA complex and free RNA were quantified and analyzed by GraphPad Prism 4. Binding percentages from three measurements were calculated to derive the apparent *K*_d_ between cTSN and RNA by a one-site binding curve fitting using the equation *Y* = *X*/(*K*_d_ + *X*), where *X* is the cTSN concentration and *Y* is the percentage of bound RNA/total RNA.

### RNA degradation assays

For the RNA degradation experiments shown in Figure 2A, pre-miR142 (sequences listed in Fig. 3) was labeled at the 5′ end with fluorescein (Dharmacon). The 5′-end fluorescein-labeled pre-miR142 (500 nM) was incubated with cTSN (100 nM) in reaction buffer containing 50 mM Tris–HCl (pH 7.6), 100 mM NaCl, 5% glycerol, 0.01% CHAPS, and 5 mM β-mercaptoethanol at 25°C for 1 h with or without the presence of different metal ions. The RNA degradation results were analyzed by 10% TBE–PAGE.

For the RNA degradation experiments shown in Figures 4 and 5, the 5′-fluorescein-labeled RNA (500 nM) was incubated with cTSN (100 nM) or the cTSN mutants (100 nM) in reaction buffer containing 50 mM Tris–HCl (pH 7.6), 100 mM NaCl, 5% glycerol, 0.01% CHAPS, 5 mM β-mercaptoethanol, and 1 mM CaCl_2_ at 25°C for 1 h (longer incubation time of 3 h was used for Fig. 5B). The cleavage results were analyzed by 10% or 15 % TBE–PAGE.

### SAXS

Small-angle X-ray scattering data were recorded at 15°C at the SAXS beamline 23A NSRRC in Hsinchu, Taiwan. The osTSN protein sample (1 mg/mL in PBS buffer containing an additional 150 mM NaCl and 5 mM β-mercaptoethanol at pH 7.4) was injected into an Agilent-Bio SEC-3 column at a flow rate of 0.02 mL/min. An X-ray wavelength of 0.8266 Å was used for data collection. Selected frames were merged and analyzed for initial *R*_g_ estimation by the PRIMUS program (Konarev et al. 2003), and then by the GNOM program (Svergun 1992) for *D*_max_ and *P*(*r*) distance distribution (ATSAS program suite, version 2.7) (Petoukhov et al. 2012). Computation of the ensemble of osTSN was conducted using the Ensemble Optimization Method using ATSAS online (Tria et al. 2015).
